# A Review of Domain Modelling and Domain Imaging Techniques in Ferroelectric Crystals

**DOI:** 10.3390/ma4020417

**Published:** 2011-02-16

**Authors:** Prashant R. Potnis, Nien-Ti Tsou, John E. Huber

**Affiliations:** Department of Engineering Science, University of Oxford, Parks Road, Oxford, OX1 3PJ, UK; E-Mails: Prashant.Potnis@eng.ox.ac.uk (P.R.P.); Nien-ti.Tsou@eng.ox.ac.uk (N.T.T.)

**Keywords:** single crystals ferroelectrics, microstructure, characterization techniques

## Abstract

The present paper reviews models of domain structure in ferroelectric crystals, thin films and bulk materials. Common crystal structures in ferroelectric materials are described and the theory of compatible domain patterns is introduced. Applications to multi-rank laminates are presented. Alternative models employing phase-field and related techniques are reviewed. The paper then presents methods of observing ferroelectric domain structure, including optical, polarized light, scanning electron microscopy, X-ray and neutron diffraction, atomic force microscopy and piezo-force microscopy. Use of more than one technique for unambiguous identification of the domain structure is also described.

## 1. Introduction

After the discovery of dielectric hysteresis in Rochelle salt by Valasek [1], the study of ferroelectric crystals expanded into a major research field and numerous applications followed. Among the main applications [2] are capacitors that exploit the high dielectric constant, transducers using the piezoelectric and pyroelectric effects, optical components with electro-optical, birefringent or scattering properties, and memory devices based on the ferroelectric remnant polarization.

Two major strands of materials research related to ferroelectric crystals can be identified. Firstly, materials development focuses on processing and characterisation of ferroelectrics to achieve the properties needed in practical applications. This has led to the discovery of compositions with significant ferroelectric hysteresis, processing techniques that enhance the piezoelectricity or polarization of poled ferroelectrics, and characterisation techniques to measure material properties. Through developments in processing it is also possible to produce ferroelectric materials in a variety of forms such as bulk polycrystals, single crystals, thick and thin films or nanodots.

A second strand of research is directed towards understanding and modelling the structure of ferroelectrics—crystal parameters, domain structure, microstructure and so forth. Until recent years, the study of domain structure informed the development of new models and applications but was not the key factor leading these developments. Major developments in piezoelectrics in the last two decades, such as the use of phase transformations in single crystals to achieve enhanced piezoelectric strains [3], and the development of strongly coupled lead-free piezoelectric ceramics [4], were experimentally led. However, in both cases, the discoveries rely on particular features of microstructural arrangement. In the case of ultrahigh strains in PMN-PT and PZN-PT, an engineered domain configuration optimises the contribution of the rhombohedral-tetragonal phase change to straining. Similarly, the strong piezoelectric effect in lead-free ceramics relied on a highly textured microstructure. Cohen [5] observed that, in the near future, predictive theory could lead the discovery of new ferroelectric materials. While this observation referred mainly to the role of first-principles methods in understanding strong electromechanical coupling, recent advances in understanding and modelling microstructure may also enable tailored material properties by design. In this article, we review theoretical descriptions of ferroelectric domain patterns and their evolution. Various techniques used for observing the domains in ferroelectrics are also discussed. The emphasis is on bulk single crystals; however application to thin films and nano-scale devices is also discussed.

## 2. Domain Modelling in Ferroelectric Crystals

### 2.1. Crystallography and Ferroelectric Domains

**Figure 1:**
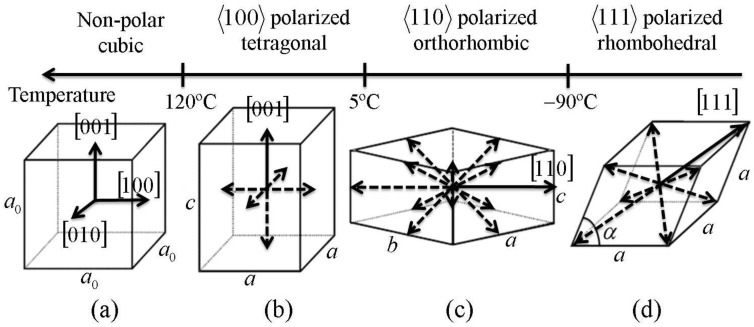
Phase transformations in barium titanate (Shu and Bhattacharya [6]). **(a)** cubic crystal system; **(b)** tetragonal system with 6 crystal variants; **(c)** orthorhombic system with 12 variants; **(d)** rhombohedral system with 8 variants.

Ferroelectric crystals are defined by having a spontaneous polarization, that can be reoriented by an electric field [2]. The spontaneous polarization is induced by a non-centrosymmetric crystal structure that is stable over some temperature range. For example, barium titanate (BaTiO_3_) is in a paraelectric phase with no net polarization above the Curie temperature (T_c_ = 120 °C), but adopts a polar tetragonal phase in the temperature range 5 °C to 120 °C. The polar tetragonal phase has 6 stable polarization directions parallel to the edges of the unit cell, resulting in 6 distinct crystal variants. Figure 1 shows the various phases adopted by barium titanate over a range of temperatures [6].

These crystal structures are commonly found in perovskite ferroelectrics and are significant in that they determine the set of available polarization directions. At the microstructural level, regions with uniform electrical polarization form domains. Thus, a domain is a region of crystal in which only a single crystal variant is found. Wherever domains meet, thin interfaces known as domain walls form [7]. Ferroelectric crystals can adopt a stable, minimum-energy arrangement of domains and domain walls, consistent with their boundary conditions, such as the overall average strain and polarization states caused by imposed displacements and charges at the crystal surfaces. However, in many cases, a unique global minimum cannot be achieved and the stable state is only a local energy minimum. Energy minimization results in crystals consisting of multiple domains, separated by domain walls. The domain walls have well-defined orientations that minimize energy by maintaining compatibility of strains and polarizations across the wall. Thus, particular patterns, or domain structures, occur and these dictate the effective properties of the crystal. If a model is to represent the material behaviour accurately, it must have the ability to describe the influence of domain structure and its evolution.

### 2.2. Micromechanical Switching Models

During manufacture, the process of cooling through the Curie temperature induces spontaneous polarization in crystallographically favoured directions. However, the random nucleation of domains typically results in a state of zero average polarization and zero residual strain. By applying an electric field, the polar direction of the unit cells can be forced into alignment with the field. This reorientation of spontaneous polarization, known as ferroelectric switching, also induces straining of each unit cell, leading to a macroscopic change in the dimensions and net polarization of the crystal. Many models of ferroelectric switching have been developed.

A key feature in switching models is that the ferroelectric crystal is treated as a material containing regions with different polarized states [8]. The model developed by Hwang *et al*. [9] is an early example of micromechanical modelling, wherein a polycrystal is represented by many randomly oriented single crystal grains. Uniform stress is assumed throughout the entire polycrystal, and each grain contains just one crystal variant. The central idea of this model is that switching occurs when the work done by local electro-mechanical fields exceed a critical threshold. Similar concepts are used and extended in other models [10,11,12]. A natural extension is to model grains containing multiple domains, and allow incremental switching of material between domain types [13]. This can provide a smoother and more accurate prediction of hysteresis response [8]. In polycrystal models, various methods have been employed to account for the inhomogeneity of stress and electric fields. At the simplest level, the Reuss approximation of uniform stress and electric field [9,10,12] neglects inhomogeneity, while self-consistent theory [13,14,15] estimates the interaction between grains and their surroundings using the Eshelby inclusion method. Finite element studies [16,17,18,19,20] allow detailed computation of the fields in each grain at the cost of computational resources. Further details of micromechanical switching models can be found in several review papers [8,21,22,23].

Models of polycrystals commonly exploit the randomness of the microstructure to smear out the material response. By contrast, single crystals show ordered patterns of domains that affect the overall behaviour. By “domain engineering” stable domain structures can be formed that enhance the electromechanical properties and performance [3,24,25,26,27,28,29]. Domain structure in single crystals also strongly influences the ferroelectric hysteresis, coercive field and remanent polarization [30,31,32,33,34]. Thus modelling of both the small field and large field properties of single crystals should take into account the domain structures that form in the crystal. In the following section, we review models of minimum-energy domain structure and discuss the consequences for the evolution of domains under load.

### 2.3. Theory of Domain Compatibility

At an equilibrium state of a ferroelectric crystal, the total of the energy stored in free-space and in distortion of the crystal, the potential energy of the external loads, and the domain wall energy is minimized [6]. As a consequence, the applied loads favour particular crystal variants that align the polarization with the external electric field and match the lattice strain to the applied stress. A further consequence is the formation of compatible domain walls. These have continuity of lattice strain and no net charge (continuity of the normal component of electric displacement). Theories of domain compatibility in ferroelectrics and related materials, such as magnetoelastic solids, have been developed by many researchers [6,27,35,36]. For a pair of ferroelectric domains i and j with lattice strain states $\varepsilon_{i}$, $\varepsilon_{j}$, and corresponding polarization vectors $p_{i}$, $p_{j}$, the interface normal vector n of a compatible domain wall must satisfy:

(1) $$\varepsilon_{i}-\varepsilon_{j}=\frac{1}{2}\left(a⊗n+n⊗a\right)$$

(2) $$\left(\;p_{i}-p_{j}\right)\cdot n=0$$

Provided a non-trivial vector a exists that satisfies Equation (1), there is compatibility of strains. Equation (2) ensures continuity of electrical polarization, giving a charge-free domain wall in the absence of electric field or stress.

**Figure 2:**
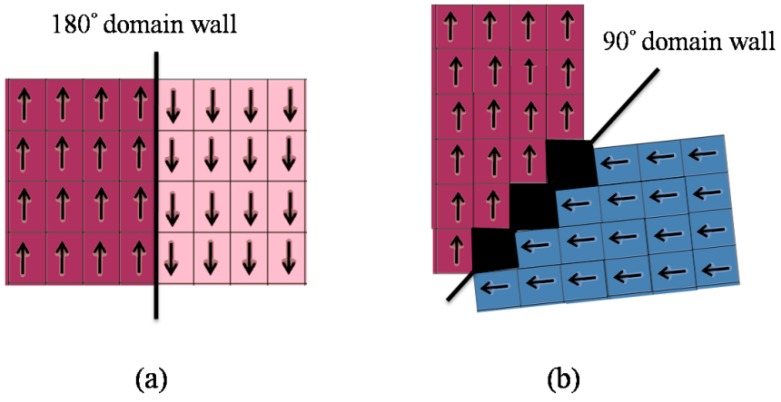
**(a)** A 180° domain wall separating lattices with anti-parallel polarizations; **(b)** A 90° domain wall.

We can examine compatible domain wall orientations for different crystal systems by solving Equations (1) and (2). For example, there are two types of domain wall in the tetragonal crystal system: 180° and 90° domain walls. Figure 2a shows a 180° domain wall separating regions of crystal lattice with anti-parallel polarizations and identical strain states. Figure 2b shows a 90° domain wall, across which the polarization rotates through about 90°. In this crystal system, the compatibility conditions give a unique domain orientation for each 90° domain wall, while 180° domain walls have no such habit plane. The 180° domain walls may thus meander through the crystal, producing commonly observed “watermark” patterns in ferroelectric crystals [7]; this can produce a non-unique minimum energy configuration. Similarly, orthorhombic crystals produce 60°, 90°, 120°, and 180° domain walls while rhombohedral crystals have 70.5°, 109.5°, and 180° domain walls [6,24].

A common form of domain pattern in ferroelectric crystals is a laminate of alternating domains [27,37,38]. In energetic terms such a domain pattern is the result of a competition between the reduction in energy achieved by mixing two types of domain (thus improving alignment of the average polarization with the external field) and the energetic cost of the domain walls. The competition of energies determines an equilibrium domain wall spacing. It is also possible that the minimum energy state consists of several such laminates, sandwiched together to form a multi-rank lamination [6].

**Figure 3:**
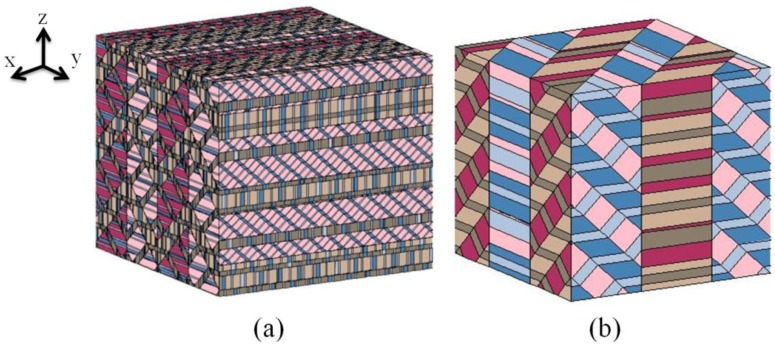
Schematic domain structures in a tetragonal crystal with all 6 types of domain present. **(a)** A rank-5 arrangement with average compatibility **(b)** A simpler, rank-3 arrangement with exact compatibility [41].

Li and Liu [27] developed a model of ferroelectric domain structure based on lamination theory, following the work of Bhattacharya [39] and DeSimone and James [35]. This approach treats the domain pattern as a periodic, multi-rank laminate of domains in which compatibility requirements are satisfied at each level of lamination, giving a low energy structure overall. An appropriate construction [27] guarantees a compatible domain structure for any feasible state of average strain and polarization. However, since the compatibility conditions are satisfied only in a volume average sense, this allows some local incompatibilities between sub-laminates. The resulting structure is then not an energy minimizer unless it forms a fine mixture [36] with a separation of length scales between successive laminations. Then the sub-laminations are taken to be sufficiently fine that the resulting laminate can be treated as a homogeneous medium. If n distinct crystal variants coexist, the construction used by Li and Liu [27] requires n−1 levels of lamination, producing extremely fine domain structure. For example, Figure 3a shows a very complex rank-5 laminate of six types of domain (six distinct colours) in a tetragonal crystal following the construction of Li and Liu [27].

An alternative approach is that of exactly compatible domains. This means that every domain wall satisfies the compatibility Equations (1) and (2), which greatly restricts the possible patterns. Several examples of exactly compatible domain patterns are described in the literature [6,7,38,40,41]. Rodel [40] includes a discussion of the effective material properties with and without local incompatibility. Tsou and Huber [41] describe a procedure for finding exactly compatible laminate structures of minimum rank for a given state of average strain and polarization. An example of this is shown in Figure 3b; this laminate has identical average strain and polarization to the structure shown in Figure 3a. However, the domain structure shown in Figure 3b has one-to-one perfect alignment of compatible domains. It is a rank-3 laminate, which is the least rank possible to produce this particular state of strain and polarization.

A complication arises due to the finite lattice shear strain between distinct crystal variants. This has the consequence that some arrangements of domains produce a disclination in the crystal lattice. In barium titanate, for example, the tetragonality of the unit cell is close to 1%. Thus the true rotation of the polarization vector across a 90° domain wall is 90.62° [6]; this effect is shown (exaggerated) in Figure 2b. Since the lattice planes turn by 0.62° across each domain wall, a disclination exists at the junction of four 90° domain walls. Where such groups of domains meet, the requirement for continuity of the crystal lattice imposes a state of stress at the domain junction [42]. Savytskii and Bismayer [43] provide a condition for strain free configurations of domains meeting at a line. A similar condition was given by Shu and Bhattacharya [6]; Tsou and Huber [41] performed a systematic search for low-rank compatible laminates, demonstrating that several commonly occurring laminate structures of rank-2 are disclination-free.

### 2.4. Domain Evolution Models

In ferroelectrics, the domain pattern greatly influences the switching behaviour, motion of domain walls and electromechanical hysteresis response. Models which take domain structure into account fall broadly into two types, diffuse interface and sharp interface, depending on how the domain wall is represented. Diffuse interface models treat the domain walls as part of a continuum with the polarization varying continuously through the wall. Among diffuse interface approaches, the phase field method is one of the most commonly used techniques. Atomistic calculations such as *ab initio* models also have great potential to model equilibrium domain structures [44,45,46]. By contrast, in sharp interface models there is a jump in polarization at the domain wall, and the detailed structure of the wall is neglected. In this section, several phase field and several sharp interface models are reviewed and compared.

#### 2.4.1. Phase Field Models

A phase field model describes ferroelectric domain patterns by using an order parameter that takes on distinct values in the different domains [47]. Various choices of order parameter are possible, but the chosen parameter must be able to discriminate among the set of crystal variants. For example, the local polarization is a convenient choice of order parameter, as it usually takes on a distinct value in each crystal variant. Then a region with uniform polarization has uniform order parameter, representing a single domain, and the transition region between a pair of domains has a continuously varying order parameter, indicating a domain wall. A major advantage of this method is that it requires no prior assumptions of domain structures which might form [48]. However, phase field models [32,48,49,50,51,52,53,54,55] must resolve the domain wall, which is commonly of order nanometres in thickness. Thus, where discretization is used, many elements are needed to simulate regions of microstructural scale.

The evolution of domain structure from a non-equilibrium state towards an equilibrium state reduces the free energy, consisting of the bulk free energy, domain wall energy, electrostatic and elastic energy, and the potential energy of applied loads. These energies can be expressed in terms of the chosen order parameter. For example, the domain wall energy arises from gradients of the order parameter, while the bulk free energy is typically a multi-well function of the order parameter. Thus, by minimizing the total energy, equilibrium states of the order parameter can be found. The main differences between phase field models are in the treatment of various contributions to the total energy expression [47] and the choice of order parameter.

The minimisation of free energy including both a gradient term and stored energy that is a function of the order parameter can be achieved by a relaxation method, with linear kinetics. This leads to an evolution law in the form of the time-dependent Ginzburg-Landau (TDGL) equation [56,57]. Models using this theory usually choose the polarization as the primary order parameter [32,48,51,58,59], with most adopting periodic boundary conditions for the convenience of computation. These models have been applied to study a wide variety of problems related to ferroelectric microstructure. Cao and Cross [58] studied the twin structure and domain wall orientation in the tetragonal crystal system. Hu and Chen [51] have successfully modelled the transformation between cubic and tetragonal phases in bulk barium titanate. Wang *et al*. [59] reveal the evolution of domain structure during switching under electromechanical loads. Zhang and Bhattacharya [60,61] solve explicitly for the electrostatic potential and thereby study non-periodic domain structure. More recently, Choudhury *et al*. [48] studied the relationship between the value of the coercive field and the presence of different types of domain in bulk ferroelectric crystals. Su and Landis [32] investigated the electromechanical pinning effect of charges on 180º and 90º domain walls. Using the same model, Kontsos and Landis [62,63] further investigated pinning by dislocations, and the formation of domain structure in thin films.

Choices of order parameter other than the local polarization have also yielded valuable insights. Shu *et al*. [53] introduce the concept of hierarchical laminate structures into their model and include the volume fractions of laminates as order parameters in addition to polarization. The resulting phase field model makes the well structure of the free energy more explicit, reducing the number of fitting parameters required. The method has been used to study stable periodic domain patterns [64].

**Figure 4:**
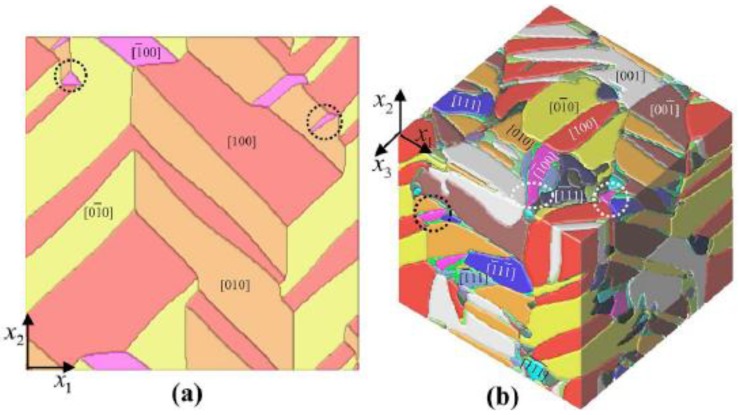
Domain structures of a PZT single crystal obtained from the phase field model by Choudhury *et al.* [48]. The polarization directions of the distinct phases and domain types are shown in different colours. **(a)** Domain structure from the 2D simulation; **(b)** Domain structure from the 3D simulation containing both rhombohedral and tetragonal phases.

We take the work of Choudhury *et al*. [48] as an example of a typical phase field model. The model is used to simulate the evolution of domain structure in 2 and 3-dimensions (see Figure 4), and reveals the influence of dimensionality on the coercive field of a bulk PbZr_*1−x*_Ti_*x*_O_*3*_ (PZT) single crystal [48]. The model also shows nucleation of new domains from existing domain walls. The spontaneous polarization P(r,t) is chosen as the order parameter, where r is the position vector and t is time. The total free energy in the crystal with volume V is given by

(3) $$F=\int_{V}(f_{bulk}+f_{elas}+f_{grad}+f_{elec})dV$$

where $f_{bulk}$, $f_{elas}$, $f_{grad}$, and $f_{elec}$ are the bulk free energy density, the elastic energy density, the gradient energy density, and the electrostatic energy density, respectively.

The bulk free energy density $f_{bulk}$ is set as a function of polarization, producing a well structure that can describe the morphotropic phase boundary compositions of PZT with 14 wells corresponding to the tetragonal and rhombohedral phases. The $f_{bulk}$ term has a local minimum whenever the polarization aligns to a rhombohedral or tetragonal crystal variant. The elastic energy density $f_{elas}$ is a function of the elastic strain, which is the difference between the total strain of the crystal and the spontaneous strain; this is assumed to be quadratically related to the polarization. Minimization of $f_{elas}$ thus drives material towards the spontaneous strain state corresponding to the current polarization. The gradient energy density $f_{grad}$ is proportional to the square of the magnitude of polarization gradient, which is nonzero only near domain walls. Finally, the electrostatic energy density $f_{elec}$ accounts for dipole interactions, depolarization fields due to surfaces, and the applied electric field. Evolution towards the equilibrium state can be described by the time-dependent Ginzburg-Landau equation:

(4) $$\frac{\partial P(r,t)}{\partial t}=-L\frac{\partial F}{\partial P(r,t)}$$

where L is a kinetic coefficient related to the mobility of domain walls. By solving Equation (4) the details of the evolution under the given loads can be determined. Choudhury *et al*. [48] simulated the evolution of domain structure under elecromechanical loads and predicted the corresponding dielectric hysteresis loops. They concluded that the presence of multiple types of domain has significant effect on the value of the coercive field in bulk ferroelectric crystals. An example of the complex 3-dimensional structures that were predicted is shown in Figure 4b.

#### 2.4.2. Sharp Interface Models

Sharp interface approaches [11,33,34,65,66,67,68] treat a domain wall as a discontinuity, across which the polarization and strain may jump. A common approach is to treat the domain wall as a crystal defect and find the driving force for motion of that defect, for example through the use of the Eshelby energy momentum tensor [65,69,70]. Motion of domain walls can thus be predicted, and equilibrium domain arrangements obtained. It is often convenient to assume particular domain topologies in models of this type. If flat domain walls are assumed, simple evolution laws can be found [33,34,66,67]. When considering periodic structure, it can be convenient to treat the volume fractions of the crystal variants as thermodynamic variables [67,68]. Alternatively, the positions of individual domain walls [33,34,66] or length of a growing feature [65] may be used as the variables. The use of the Eshelby energy momentum tensor requires knowledge of local fields that can be derived by solving for the equilibrium of the system in its current configuration. Alternatively, a global approach may be used, in which a global potential is minimised. In either case a kinetic relation is needed to infer the rate of domain wall motion from the driving force. Linear kinetics are commonly assumed, and this introduces the domain wall mobility as a factor governing the rate of domain wall motion.

Loge and Suo [65] formulate a kinetic model using a functional containing the rate of change of free energy and a dissipation potential. In their work, the evolution of a one degree of freedom domain stripe and an elliptical domain region with two degrees of freedom were studied. Similarly, Huber and Cocks [66] use a variational principle, previously applied to a variety of problems in microstructure evolution [71], to model the hysteresis response of BaTiO_3_. In their model, a domain pattern with two degrees of freedom and linear kinetics were assumed. Yen *et al*. [68] combine the concept of a switching criterion and compatible laminate theories [27] to model the hysteresis response of BaTiO_3_, assuming a multi-rank averagely-compatible laminate structure. Similar work by Weng and Wong [67] develops a thermodynamic framework for specific rank-1 and rank-2 compatible domain laminates, such as the commonly observed herringbone pattern [7]. Their model predicts the hysteresis response of BaTiO_3_, showing features in common with the results of experiments by Burcsu *et al*. [72]. The kinetic model developed by Tsou and Huber [33] studies the evolution of particular domain patterns such as vortex arrays and herringbone structures under electromechanical loads. Stable equilibrium states of each topology are obtained. In further work [34] nucleation of new domain topologies is allowed so that one laminate pattern can evolve into another, through a shared “pivot” state.

**Figure 5:**
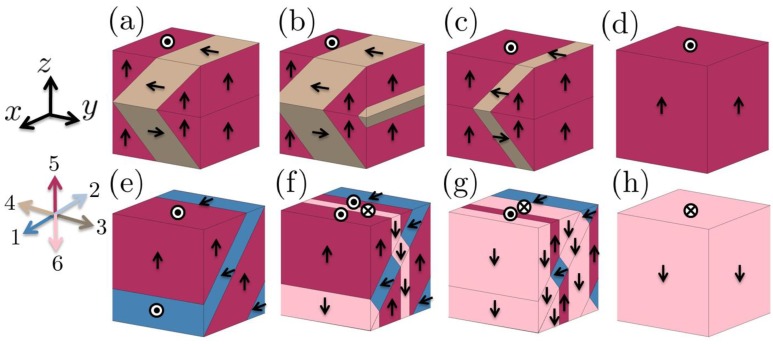
A schematic domain evolution when a BaTiO_3_ crystal subjected to a cyclic electric field and a small compressive stress along *z* direction, where the polarization directions of crystal variants numbered from 1 to 6 [34].

Let us take the sharp interface model developed by Tsou and Huber [34] as an example of sharp interface modelling. The domain topologies considered are periodic rank-2, exactly compatible herringbone patterns. Two examples of such rank-2 herringbone domain topologies are shown in Figure 5a and Figure 5f, where the polarization directions of tetragonal crystal variants are numbered from 1 to 6 and indicated using distinct colours. The figure shows cubical sections of a periodic structure. The concept of a “pivot state” of the domain structure can be described using the following example. Consider a rank-2 herringbone structure in a tetragonal ferroelectric, containing domains with three distinct crystal variants, numbered 3, 4, and 5, as shown in Figure 5a. Let the 90º domain walls move, so as to change the volume fractions of the variants while keeping the same topology (Figure 5b, c). When domains with variants 3 and 4 disappear altogether, the structure becomes a single domain (Figure 5d). From this single domain state, a new topology of domains can nucleate if it is energetically favourable. For example the single domain state can evolve into a laminate of crystal variants 1 and 5 as shown in Figure 5e. The single domain state here served as a “pivot state” enabling a transition between distinct forms of domain structure.

The kinetic framework for domain evolution [34] uses the domain wall positions $a_{i}$ for the *i*th domain wall as thermodynamic variables. For example, in the periodic domain structure of Figure 5a there are two degrees of freedom, corresponding to the positions of the 90° and 180° domain walls. These variables are used to express the energy terms in a functional Π defined by:

(5) $$\Pi =\dot{G}+\Psi$$

where G is the total Gibbs free energy which is the sum of the internal stored energy and potential energy due to the loads, and Ψ is a dissipation rate associated with the area, velocity, and mobility of domain walls. It is readily shown [65] that the domain structure evolves along a path which makes the functional Π stationary with respect to the rates of the degrees of freedom ${\dot{a}}_{i}$, that is:

(6) $$\frac{\partial \Psi }{\partial {\dot{a}}_{i}}=-\frac{\partial G}{\partial a_{i}}$$

The domain wall velocities ${\dot{a}}_{i}$ can then be obtained directly by solving Equation (6). The assumption of linear kinetics leads to a particularly simple form in Equation (6).

Figure 5 [34] shows a prediction of domain evolution in BaTiO_3_ with a monotonically increasing electric field and a constant compressive stress along the z direction. As the electric field is cycled, there is first 90° switching (Figure 5a-d) followed by mixed 90° and 180° switching (Figure 5e-h) after the nucleation of a new herringbone domain topology.

### 2.5. Models for Ferroelectric Films

The models discussed so far focus on bulk ferroelectric crystals; however, thin film devices have also been studied extensively in the last decade. Ferroelectric films have many advantages in applications, such as small size, low operating voltage, high speed, and ease of production for materials that are difficult to produce in bulk [2]. Furthermore, ferroelectrics films have great potential for domain engineering, for example by rearranging the domain orientation using techniques such as conductive atomic force microscopy [73]. This can create new structures with attractive properties [74]. Alternatively, periodic structures in films can be used to provide a template for a patterned device [75].

In ferroelectric films, both the crystallographic orientation and the strain state of the film are strongly influenced by the substrate. This dramatically affects the properties of the film, such as Curie temperature and microstructure [76]. For example, when a BaTiO_3_ film is subjected to a biaxial compressive substrate strain, its cubic-to-tetragonal transition temperature can be increased by as much as 500 °C relative to a bulk single crystal [77]. The substrate constraint also imposes a state of in-plane strain that governs the stable domain structure. In sufficiently thin films, the compatibility constraints Equations (1) and (2) are relaxed in the out-of-plane direction as the deformation energy becomes much smaller than the interfacial and membrane energies [78]. Thus, a low energy elastic accommodation to out-of-plane incompatibilities is possible. As a consequence, thin films allow a greater range of low energy domain structures than bulk crystals.

In recent years, researchers have explored the switching behaviour, microstructural thermodynamics, phase diagrams, and effects of misfit strain in ferroelectric thin films [74,76,79,80,81,82,83,84,85,86,87,88]. Speck and Pompe [82] calculated the microstress due to the misfit strain and its effect on the energy of epitaxially grown films. They used temperature dependent stability maps to illustrate the behaviour of domain structures in thin films. Roytburd *et al*. [74,80] developed a thermodynamic theory based on theories of elastic domains to study the influence of the misfit of strains on the domain structure. Based on this theory, Alpay *et al*. [81] produced domain stability maps for tetragonal ferroelectric thin films. The effect of a uniaxial external stress on the domain stability maps was discussed. The related topic of martensitic transformations in constrained thin films was also studied by Roytburd *et al*. [89]. They represented the domain structure as a multi-rank laminate of different types of domain in order to calculate the overall strain states and the evolution of domain patterns. Pertsev *et al.* [83] adopt the thermodynamic calculations to give several domain stability maps for BaTiO_3_ and PbTiO_3_.

Prior thermodynamic analyses in ferroelectric films generally focused on the tetragonal crystal system and simplified the 6 types of domain orientations into 3, *i.e*., domains with parallel polarization directions were treated as identical. Then, the two types of domain with their polarization orientations parallel to the substrate surface are named *a*_1_ and *a*_2_ domains, while *c* domains contain the variant with polarization perpendicular to the film surface. Moreover, certain particular domain structures are commonly assumed, such as alternating *c*/*a*/*c*/*a* or *a*_1_/*a*_2_/*a*_1_/*a*_2_ domain patterns [79,83]. However, under certain boundary conditions, these patterns may not form as a domain arrangement with other types of domain present is energetically favourable [90]. Li *et al*. [76,84,86] used a phase field model of domain evolution in 3-dimensions without any prior assumptions of domain pattern. All three types (*a*_1_, *a*_2_, *c*) of tetragonal domain were found to co-exist and complicated structures resulted. Further details of phase field simulation for thin films can be found in the review paper by Chen [91].

Several different approaches have also been used to study the switching behaviour and domain structure in ferroelectric or other related material crystal films. Huber [85] adapts a self-consistent micromechanics model with some modifications to satisfy thin film conditions for prediction of the hysteresis response of a lead zirconate titanate (PZT) film. Similar methods were used by Pane *et al.* [92,93] to study the effects of film geometry and mechanical constraint on dielectric hysteresis. In the study of martensitic trigonal thin films, Shu and Yen [87] employ the unconventional phase field approach to investigate the formation of low energy domain patterns. Tsou and Huber [88,94] consider thicker films in which the out-of-plane compatibility conditions are still satisfied. They provide a theory of equilibrium domain structure in single crystal films and apply this theory to study the limitations placed on electrical polarization by the domain structure in both the tetragonal and rhombohedral ferroelectric crystal systems.

In this section, we have illustrated the wide variety of modelling approaches used to predict the domain structure and properties of ferroelectric crystals. We next consider the range of observation methods that can be used to evaluate such predictions.

## 3. Observation of Domain Structure in Ferroelectric Crystals

Just as there have been significant efforts to model ferroelectric domains and predict possible domain arrangements, so also the visualization of domains has been studied extensively. The goals of such study are to validate predictions of microstructure and to develop theories of material behaviour. By combining the measurement of macroscopic properties with microscopic imaging of domains an improved understanding of microstructure can be gained. The various techniques used for domain observation can be classified based on their operating principle: (1) Surface treatment techniques (surface decoration, etching) (2) Optical techniques (optical microscopy, polarized light microscopy, photorefractive methods) (3) X-ray techniques (reflection and transmission measurements, anomalous dispersion) (4) Electron microscopy techniques (Scanning electron microscopy, transmission electron microscopy and related methods) (5) Scanning probe microscopy techniques (Piezo-response force microscopy, Electrostatic force microscopy). We next discuss each of these techniques, starting from the early attempts to reveal domain structure and moving on to the most recent techniques. For each technique we highlight the capability in resolving domain structure, the limitations and any special specimen preparation or instrumentation requirements. A detailed discussion of several of the techniques can be found in the reviews of Soergel [95] and the book by Tagantsev *et al*. [96].

### 3.1. Surface Treatment Techniques

**Figure 6:**
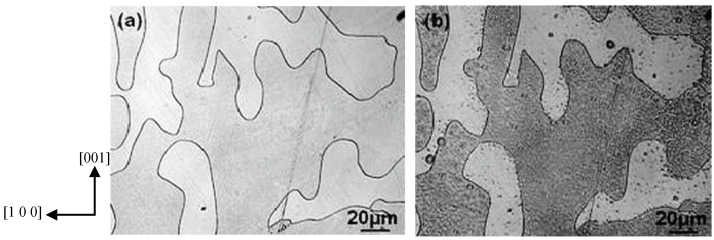
Domain structure in lithium niobate observed under optical microscope after **(a)** etching **(b)** nanoparticle decoration. (Ke *et al*., 2007 [98]).

Several early attempts to reveal ferroelectric domain structure exploited the fact that surface charges due to local polarization can interact with nearby charged or polar particles. Such surface decoration methods can use colloidal solutions, liquid crystals, or other polar particles and produce contrast or colouring of suitably oriented domains or domain walls. Hatano *et al*. [97] used a commercial liquid developer containing toner, diluted by *n*-hexane to decorate 180° domain walls in triglycine sulphate (TGS). Positively charged carbon particles in the toner were deposited on the negatively charged TGS domains. The carbon particles, of 0.1 µm diameter, coagulated to give a spatial resolution of 0.5 µm. A recent decoration method makes use of nanoparticles of polystyrene to image the domains in lithium niobate crystal wafers [98]. Figure 6 shows a comparison between an HF etched and a decorated crystal delineating negatively charged domains.

Similar observations have been made using nematic liquid crystals [99,100]. The oriented patterns of the liquid crystals were observed using polarized light microscopy. Evolution of microstructure could be observed, but this technique works best for slow processes only. It also requires a cleaved and polished crystal surface. Lateral resolution is limited by the choice of decorating medium, but resolution better than 1 µm is readily achieved.

**Figure 7:**
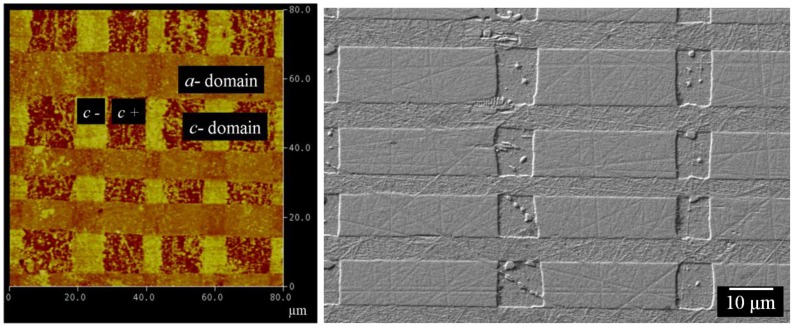
Barium Titanate single crystal etched with HF observed under **(a)** AFM (80 × 80 µm^2^ region) and **(b)** a similar region observed by SEM. Note: these images have slightly different scales [105].

A second type of surface treatment, commonly used, is etching the crystal surface using acids such as HF, HCl or HNO_3_. In barium titanate and TGS, the etching rate is fastest at the positive end of a dipole [37] while in lithium niobate and lithium tantalate etching mainly erodes the negative end of the dipole [101,102]. The surface topography produced by etching can be observed using optical microscopy, scanning electron microscopy or atomic force microscopy [103,104,105] as shown in Figure 7. Etching enables rapid and unambiguous identification of *c*-domains with sub-micron resolution [95]. However, it is a destructive technique, restricted to surfaces and does not allow in-situ observation of domain structure evolution. The technique also relies on identifying appropriate specimen preparation, etchant composition, and etching time.

### 3.2. Optical Techniques

**Figure 8:**
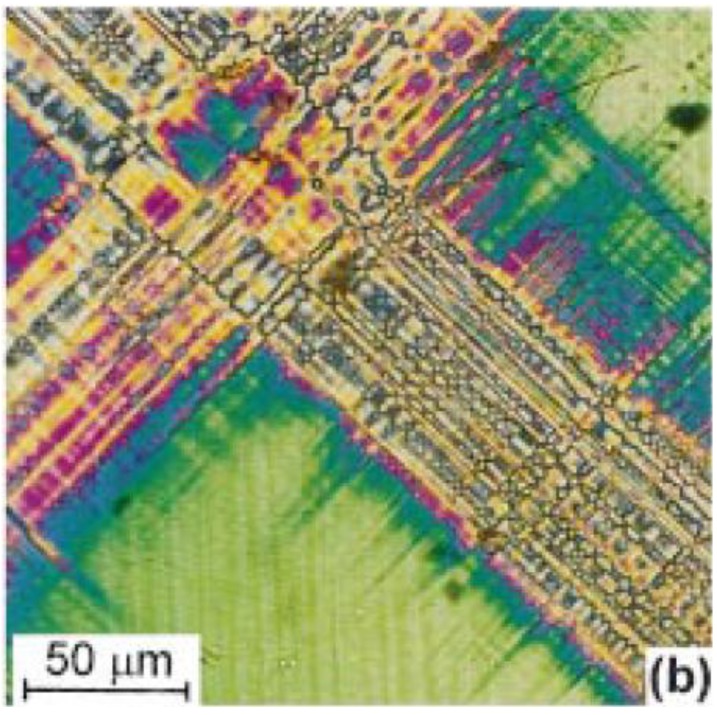
Fine lamellar domain structure in tetragonal PMN-PT observed by polarized light (Temperature = 130 °C) (Ye and Dong, 2000 [109]).

Optical methods provide simple, non-contact, imaging and allow in-situ observation of domain evolution under thermal and electro-mechanical loads. Early experiments of this type used the electro-optic effect and polarized light [106,107] to distinguish 90° and 180° domain walls under electric field. Lamellar domain structure in tetragonal lead magnesium niobate—lead titanate (PMN-PT) observed under a polarized light microscope shows mutually perpendicular domain stripes in the (110) direction (see Figure 8). Polarized light microscopy has been used for in-situ observation of domain evolution as a function of temperature in barium titanate [108], and PMN-PT [109,110]. 

A sophisticated birefringence imaging technique using rotating polarizers was developed by Glazer *et al*. [111] to automate the separation of birefringence magnitude and orientation data. Typical ferroelectrics have anisotropic dielectric permittivity, making them suitable for this technique. The method was used to show twin structure in barium titanate during the cubic-tetragonal phase transition. The technique allows full field, rapid imaging of domains and sensitivity to strains of the order of 10^−7^. Polarization microscopy of this kind provides a convenient visualization of domain structure, but is limited to transparent crystals. It also presents difficulty distinguishing surface from sub-surface structure, and resolution is typically limited to a few µm.

**Figure 9:**
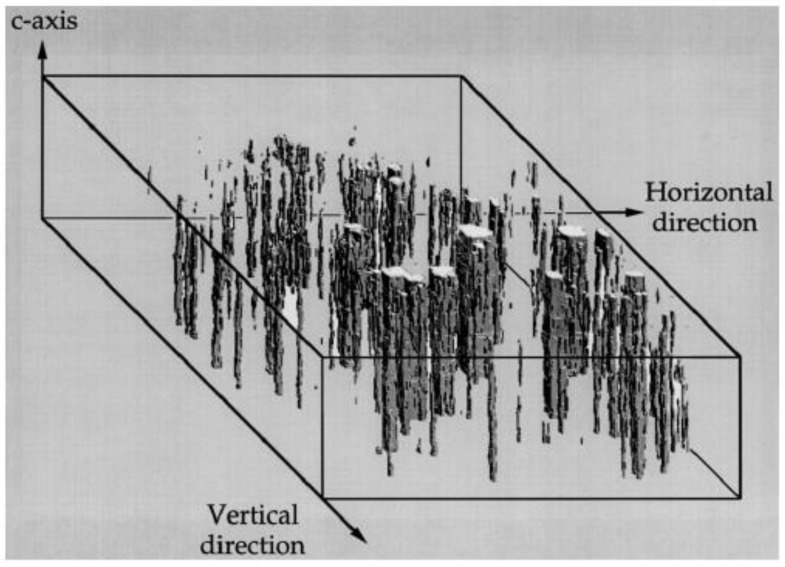
A 3-dimensional map of 180° domains in barium titanate crystals (Grubsky *et al*., 1996 [115]).

Muller *et al*. [103,112,113] used laser illumination to identify the domain walls in lithium niobate. This technique produced domain boundary images with about 10 µm resolution when the transmitted laser light was focused onto a screen. The method is based on the deflection of the laser by domain walls and can also give averaged measurements over large areas. It was used to study the domain reversal process in real time. A 3-dimensional mapping of domain structure is possible by using a photorefractive beam-coupling method [114,115]. Here, the experimental set up consists of two beams of argon laser light: a probe beam propagating along the *c-*axis of the crystal, and a pump beam intersecting the probe beam in the crystal. As the probe beam travels through c-domains, it either loses or gains energy depending on the domain orientation; providing contrast in the detected image. By scanning the position of the crystal across the pump beam, a 3-dimensional image is built up in slices as shown in Figure 9. In this work the spatial resolution was limited by the pixel size of the CCD camera to about 7µm. The technique looks promising for 3-dimensional mapping but is limited to 180° domain walls.

Second harmonic generation microscopy (SHGM) exploits the interference of second harmonic waves, produced by the difference in non-linear optical coefficients of antiparallel domains, to identify the domains present. Periodically poled domain structure in lithium tantalate (period of domains = 3.5 µm) was observed using SHGM by Kurimura and Uesu [116]. SHGM has also been used to image ferroelectric domains in TGS [117], 90° domain walls and domains in barium titanate [118] and domains walls in potassium titanyl phosphate (KTP), lithium niobate [119], and in lithium tantalate [120,121]. Recently this technique has been used to estimate the width of the domain walls in lithium tantalate to be less than 10 nm [122].

### 3.3. X-ray Techniques

X-ray diffraction techniques can identify crystal structure and domain types in ferroelectrics. The underlying principle is the detection of the distinct lattice parameters: this can readily distinguish, for example, between *a*-domains and *c*-domains in barium titanate. However, where there is no change of lattice parameter, such as across a 180° domain wall, refinement of the technique is needed. Anomalous dispersion of X-rays causes a difference in the intensity of reflections between antiparallel domains. This method was used by Niizeki and Hasegawa [123] to observe antiparallel 180° domains in barium titanate single crystals. Park *et al*. [124] carried out white beam X-ray topography of barium titanate single crystals to observe the domains and strain fields.

Antiparallel ferroelectric domains in barium titanate single crystals were observed by Fogarty *et al*. [125] using high resolution X-ray diffraction imaging with monochromatic light. The technique revealed domains in the interior of a 1 mm thick specimen with a spatial resolution of about 1 µm. Use of a synchrotron X-ray source in these experiments enabled visualising large specimen areas without multiple scans and observations in Laue geometry (transmission topography) to image domains in the interior of the crystal. Fogarty *et al*. also used Bragg geometry (reflection topography) to map the domain structure on the surface of these crystals and showed that the interior domain structure differs significantly from the surface structure.

**Figure 10:**
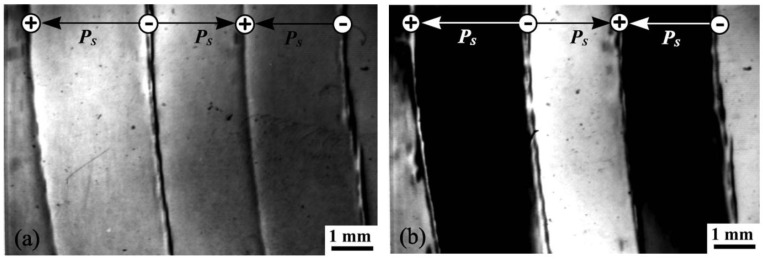
Contrast intensification in the [030] reflection of a lithium niobate crystal with **(a)** no electric field; and **(b)** electric field applied. (Roshchupkin *et al*., 2009 [126]).

Domain structure in lithium tantalate and lithium niobate has been studied under the application of electric field using high resolution X-ray diffraction [126]. Application of electric field produces deformations of opposite sign in the antiparallel domains due to the converse piezoelectric effect, increasing the contrast in Bragg reflections (Figure 10). The method has the potential to map *in-situ* domain evolution under electromechanical loading.

Lattice distortions in the vicinity of 90° and 180° domain walls in several ferroelectric crystals were measured using X-ray diffraction [127,128,129]. These works suggest that the residual strain field of the domain wall extends several µm from the walls. More recently, domain switching in rhombohedral PZT was studied using in-situ high energy synchrotron X-ray diffraction by Hall *et al*. [130]. The high flux and energy available from synchrotron X-ray sources allows mapping domain structure both on the surface as well as in the interior of the crystal. The resolution achieved is limited by the detectors and the quality of the light source.

Neutron diffraction techniques have the advantage of penetrating the full specimen thickness and thus give statistical information about lattice spacing and orientation over the specimen volume. This has been used with in-situ loading to examine polarization reversal in lead zinc niobate—lead titanate (PZN-PT) [131], texture and lattice strain studies in PZT [132,133], phase transformations in PZT ceramics [134]. Collection times are typically greater than those for X-ray diffraction, and lower lateral resolution is achieved. Further discussion of the use of X-ray and neutron diffraction on ferroelectric materials is given in the review by Jones [135].

### 3.4. Electron Microscopy Techniques

Imaging of domains using scanning electron microscopy (SEM) is challenging as ferroelectrics are non-conducting, leading to charge build-up on non-metallized surfaces. However, ferroelectric surfaces can be directly imaged in secondary electron mode using low acceleration voltages. Contrast between domains can arise through electrostatic interactions between the specimen and electron beam [136], wherein electrons are attracted to the positive end of the dipole.

**Figure 11:**
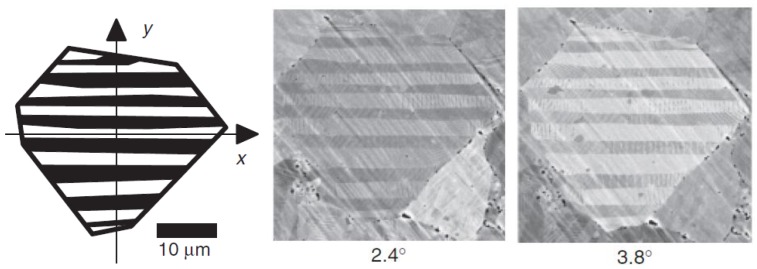
Back-scattered electron images showing effect of specimen tilt on domain observation in (Na,K)NbO_3_ (Gruner and Shen, 2010 [137]).

This contrast is not normally visible in back-scattered electron (BSE) mode, and is highly sensitive to use of the correct accelerating voltage. However, BSE mode can be used to image the domain structure in ferroelectrics by exploiting the contrast due to electron channelling that depends on the tilting of the domains [137]. Figure 11 shows effect of specimen tilt in imaging domains in (Na,K)NbO_3_ using BSE imaging. Domain boundaries can also be revealed by the converse piezoelectric effect due to the electric field generated from charge build-up at the specimen surface. Rosenman *et al*. [138] used this method to observe domain and boundary contrast in KTP.

The use of environmental-SEM (ESEM) alleviates charge build-up, enabling greater acceleration voltages. Then the pyroelectric potential induced by local heating becomes a possible mechanism for domain contrast. Zhu and Cao [139,140] observed anti-parallel domains in cleaved and polished lithium tantalate in this way. The domain structure observed by ESEM on the polished surface correlated well with observations by the etching technique. Scanning electron microscopy is a rapid technique with sub-micron resolution, but surface charging and surface damage can interfere with measurements. The need for vacuum makes in-situ domain evolution experiments relatively difficult.

Electron Back Scatter Diffraction (EBSD) gives the local crystallographic orientation at points within an SEM image. This technique has been used for mapping herringbone domain structure in bismuth ferrite-lead titanate single crystals [141]. A similar technique used with lead zirconate titanate allowed estimation of the lattice rotation across 180° domain walls and evaluation of the peak stress at a band junction [142]. EBSD is often used in conjunction with the other techniques to confirm the domain orientations, as discussed in the Section 3.6.

Scanning electron acoustic microscopy has also been used to map the domain structure. Here an acoustic wave generated by the converse piezoelectric effect is sensed using a piezeoelectric transducer. The signal is read through a lock-in amplifier and the phase of the signal indicates the orientation of the domains (electron acoustic image). This method, together with surface topography using secondary electrons (secondary electron image), can identify specific domains. The technique was used by Zhang *et al*. [143] to image domains in single crystal barium titanate with sufficient lateral resolution to show 5 µm domain bands clearly.

Transmission Electron Microscopy (TEM) has been used to image microdomains in barium titanate over a paraelectric-ferroelectric phase transition [144]. Hu *et al*. used bright field imaging, dark field imaging and selected area diffraction to image domains in doped barium titanate and diffraction contrast is believed to distinguish different domain types [145]. TEM has been extensively used to image the domains in number of ferroelectric crystals and it is often used to observe the changes in domain structure over a range of composition, for example, in PMN-PT solid solutions [146], PZT solid solutions [147] and tungsten bronze ceramics [148].

High resolution transmission electron microscopy (HRTEM) has been used for the measurement of domain wall thickness in barium titanate [149,150]. The domain walls were reported to be of thickness ~5 nm or 4–10 unit cells. Similar observations in lead zirconate titanate (PZT) found the domain wall width to be between 3 and 5 nm [151]. Contrast at domain walls in the HRTEM images is attributed to lattice distortions or ionic displacements. The structure and formation of nano-twins in polycrystalline barium titanate thin films has also been observed by HRTEM [152]. Excellent lateral resolution, of the order of 1nm, can be obtained, but the preparation of thin samples, typically 10 μm or less, is vital.

### 3.5. Scanning Probe Microscopy

Characterizing domain structure with a spatial resolution of a few nanometers is made possible by scanning probe microscopy techniques, which have revolutionized domain visualization. The Atomic Force Microscope (AFM) is the key instrument underlying scanning probe techniques. This is an extensive field of study and separate reviews by Bonnell [153] discussing the origins of AFM, and Kalinin [154,155], Gruverman and Kholkin [156] on applications to ferroelectrics, provide broad coverage. The AFM is typically operated either with the tip in contact (repulsive force regime) or in non-contact (attractive force regime). Lift or interleave mode can also be used, in which a surface is first scanned in contact and then rescanned with the tip lifted to a predetermined height. In addition to direct topographic measurement, specific modes of AFM used for detecting domain structure include electrostatic force microscopy (EFM), piezoresponse force microscopy (PFM), scanning non-linear dielectric microscopy (SNDM) and Kelvin probe force microscopy (KPFM).

It is first worth noting that domain imaging can be achieved by conventional AFM methods, without exploiting the electrical nature of ferroelectric crystals. Imaging of 90° domains can be achieved by purely topographic methods due to the shear distortion of ferroelectric crystals across 90° domain walls. This causes a surface distortion that is readily observed in AFM topographic images. For example, non-contact AFM was used by Eng *et al*. [157] to image the reorientation of the *a*-domains during the tetragonal-cubic phase transition in barium titanate. Other sources of topographic contrast include steps at domain boundaries [158] and surface corrugation [159,160]. Lateral force microscopy in contact mode can also distinguish ferroelectric domains if there is a change in surface structure between domains, leading to different friction properties. Bluhm *et al*. [161] used this method to image domain structure in triglycine sulphate (TGS).

We next turn to scanning probe techniques that exploit electrical interactions with the ferroelectric surface. In EFM, the electrostatic field of surface charges due to polarized domains is detected in non-contact mode. The interaction between the surface charge and tip charge produces a force that varies across domain walls [159]. Bluhm [162] used EFM to image a periodically poled lithium niobate crystal and found good agreement between their EFM measurements and topographic measurements. Though this method is effective in distinguishing topographic features from electrostatic effects, achieving good contrast is a challenge. A good contrast can be obtained by applying a.c. voltage to the probe tip which is also referred as dynamic contact EFM. This method is used to map the domain structure in periodically poled lithium niobate and surface deformation due to the piezoelectric effect is believed to be the reason for contrast [163]. Resolution is limited to be of the order of the distance from the tip to the surface, typically around 100nm. Surface contamination and cross-talk can cause difficulty. KPFM, also called scanning surface potential microscopy, is based on detecting the surface potential associated with a spontaneous polarization state by applying a combination of a.c. and d.c. voltages to the probe tip in non-contact mode. More detailed discussion of EFM and KPFM is given by Kalinin and Bonnell [164].

Contact mode methods that rely on electrical properties can also be effective. Scanning non-linear dielectric microscopy (SNDM) is a contact mode technique in which the sample surface acts as a capacitance in a resonant LC circuit driven by an a.c. voltage applied to the probe tip. A change in non-linear dielectric response of the sample causes change in capacitance which depends on the polarity of the domains [165]. Typically, the voltage is applied at GHz frequency and sub-nanometer lateral resolution can be achieved [166].

Perhaps the most successful of the scanning probe techniques for ferroelectric crystals is piezoresponse force microscopy (PFM). This is a contact mode technique in which piezoelectric surface deformations are generated by applying a voltage to the probe tip. Mechanical vibrations are produced due to the converse piezoelectric effect which can then be interpreted to map the local orientation of the polarization vector. In tetragonal ferroelectrics, it is possible to identify out-of-plane *c+* and *c−* domains by vertical PFM. This relies on the $d_{33}$ piezoelectric coefficient producing normal surface displacements that deflect the cantilever probe. Similarly, in-plane *a-*domains can be distinguished using lateral PFM, which exploits the $d_{15}$ piezoelectric coefficient to generate shear displacements of the specimen surface that twist the cantilever probe. In vector PFM both sets of measurements are combined to construct a map of the three dimensional polarization vector. PFM is commonly used on thin films, where moderate tip voltages are effective; however, it can also be used on bulk crystals.

**Figure 12:**
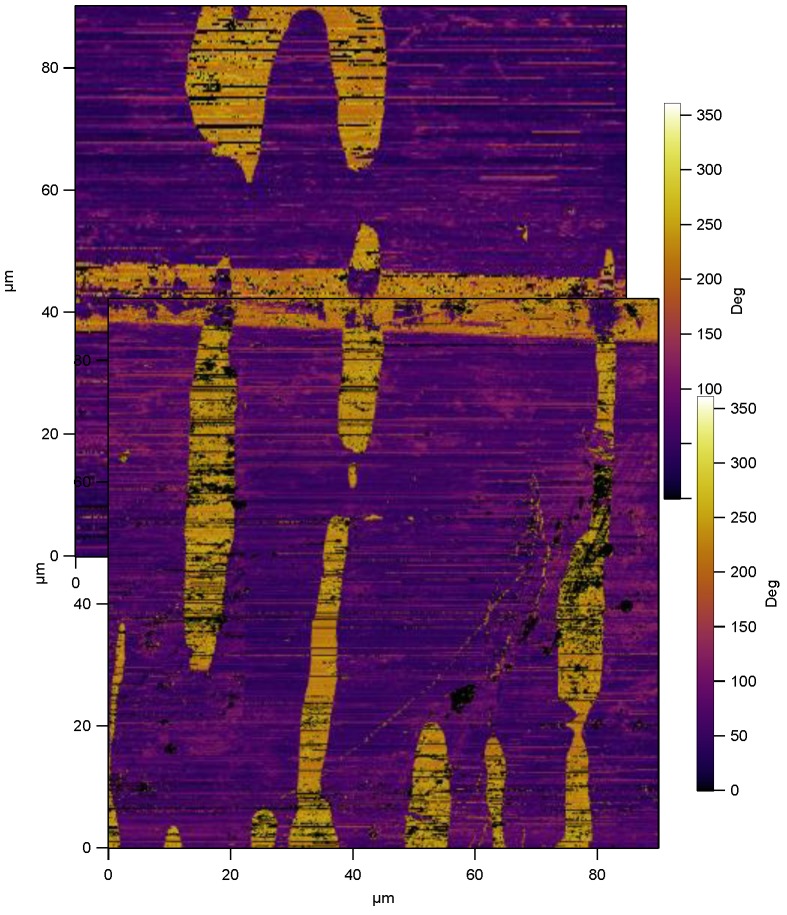
Vertical piezoresponse force microscopy on barium titanate single crystal showing *c+* and *c−* domains with 180° domain boundaries (90 µm width region).

An example of vertical PFM, is shown in Figure 12 where we image antiparallel domains in single crystal barium titanate using an MFP-3D AFM at Asylum research, UK. Surface displacements of the out-of-plane domains give a phase difference of 180° across domain walls. Figure 12 shows the phase response of the *c+* and *c−* domains, identifying the domain types and showing 180° domain walls in a watermark pattern, dissected by a straight 90° domain walls.

**Figure 13:**
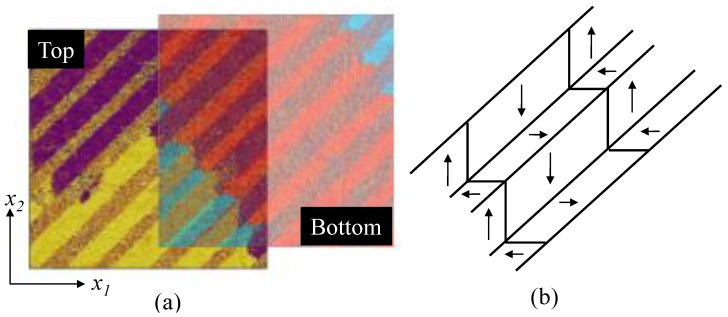
**(a)** Lateral piezoresponse force microscopy on a barium titanate single crystal showing herringbone structure (two 40µm regions), schematically represented in **(b)**.

Now, as an example of lateral PFM, consider the imaging of herringbone pattern *a*-domains in barium titanate, as shown in Figure 13(a). Here the phase of the surface displacement response is used to distinguish the different polarities of the in-plane *a*-domains. The upper part of Figure 13(a), marked “Top” shows strongly contrasting bands of domains with a 180° phase change within the band indicating 180° domain walls (purple and yellow colours). These domains are oriented such that their $d_{15}$ coefficient gives rise to surface displacements in the $x_{2}$ direction. Also visible in the “Top” image are intermediate bands of 90° domains which do not show strong contrast as their piezoelectric response produces displacements in the $x_{1}$ direction. By turning the specimen through 90°, the lower portion of Figure 13(a) was measured, marked “Bottom”. Superimposing the phase responses reveals a particular arrangement with alternate bands of 90° domains, each band containing 180° domain walls. The method unambiguously identifies the well-known herringbone structure, shown in Figure 13(b).

An important development is the use of in-situ studies to observe domain structure evolution. This is challenging due to space constraints in some AFM systems. The nucleation and growth of domains in single crystal barium titanate was observed in-situ by applying compressive stress in the work of Munoz Saldana *et al*. [160]. A similar study was carried out on lanthanum doped PZT under electromechanical loads, using small loading steps to observe the interaction between neighbouring domains [17]. PFM has also been used to study in-situ domain evolution in various compositions of PMN-PT single crystals as a function of temperature and under the application of electric field [167,168].

In summary, scanning probe methods offer extremely high (sub nanometre) resolution, and very good contrast for mapping fine domain structure. The resulting images need careful interpretation due to the possibility of cross-talk between the various effects that can give contrast. The main drawbacks of the technique are the limitation to surfaces and the limited scan area. An opportunity offered by scanning probe methods is the manipulation of domain structure using the probe.

### 3.6. Combined Methods

As the each observation method has different capabilities, more than one technique may be needed to map the microstructure and have an unambiguous interpretation of the domains present. For example, scanning and transmission electron microscopy, optical microscopy and X-ray diffraction were used to image herringbone and lamellar domain structure in barium titanate single crystals by Park and Chung [169]. Similarly, 90° *a–c* domain boundaries were imaged using polarized light microscopy, SEM and contact mode AFM in single crystal barium titanate [104]. Domain structure on the surface of a barium titanate crystal was mapped using synchrotron X-ray topography followed by SEM and contact mode AFM by Potnis *et al*. [105]. Crystallographic orientation information given by EBSD has been used to predict the vertical PFM response in polycrystalline PZT with a spatial resolution of 25 nm [170]. Similar studies can be found for the transparent ferroelectric glass-ceramic (LaBGeO_5_) [171], PZT films [172,173] and (Bi_1−x_La_x_)_4_Ti_3_O_12_ (BLT) films [174]. Domain switching along indentation cracks in barium titanate ceramic subjected to Vickers indentation was studied using X-ray diffraction in conjunction with EBSD by Cheng *et al*. [175]. By applying various techniques to the same region of microstructure, domain information can be revealed which is not available from any single technique.

Another approach to enhancing the capabilities of observation techniques is the use of modeling to predict or interpret observed domain patterns. Relatively few studies were found that give direct comparisons of modeled domain patterns and experimental imaging. Anteboth *et al*. [17] take the converse approach, using PFM imagery to define a domain pattern that is then modeled using the finite element method. Recently, Kuo et al [176] used minimum energy theory of compatible domains to interpret the observed pattern of interfaces in bismuth ferrite films. Fousek and Mokry [42] analyse observed domain patterns in potassium niobate using the theory of compatible domain arrangements, finding stressed, but minimum energy arrangements. Similarly, Potnis *et al*. [177] compare minimum energy domain patterns predicted by laminate theory with AFM observations of etched crystals. Knowledge of the possible minimum energy domain configurations aids the interpretation of observed domain patterns, particularly where there is ambiguity over domain types within the observations.

## 4. Conclusions

This article has reviewed a variety of modeling methods and characterization techniques used in the study of ferroelectric microstructure. Both the modeling and visualization of domains are rapidly developing fields of study that support each other through the prediction of, and confirmation of specific microstructural phenomena. Among modeling techniques, a compromise exists relating to computational speed: modelling from first principles remains slow for large regions of microstructure, and so phenomenological approaches that extend the size of the modelled region are necessary. Turning to the characterization techniques, different capabilities are evident, meaning that the use of multiple techniques can enhance the interpretation of data from each method. Thus both the modeler and the experimenter need a working appreciation of a wide range of techniques.
